# Relationship between mechanical load and surface erosion degradation of a shape memory elastomer poly(glycerol-dodecanoate) for soft tissue implant

**DOI:** 10.1093/rb/rbad050

**Published:** 2023-05-10

**Authors:** Kaixiang Jin, Hanqin Li, Mingkai Liang, Yuqi Li, Lizhen Wang, Yubo Fan

**Affiliations:** Key Laboratory of Biomechanics and Mechanobiology (Beihang University), Ministry of Education, Beijing Advanced Innovation Center for Biomedical Engineering, School of Biological Science and Medical Engineering, School of Engineering Medicine, Beihang University, Beijing 100083, China; Key Laboratory of Biomechanics and Mechanobiology (Beihang University), Ministry of Education, Beijing Advanced Innovation Center for Biomedical Engineering, School of Biological Science and Medical Engineering, School of Engineering Medicine, Beihang University, Beijing 100083, China; Key Laboratory of Biomechanics and Mechanobiology (Beihang University), Ministry of Education, Beijing Advanced Innovation Center for Biomedical Engineering, School of Biological Science and Medical Engineering, School of Engineering Medicine, Beihang University, Beijing 100083, China; Key Laboratory of Biomechanics and Mechanobiology (Beihang University), Ministry of Education, Beijing Advanced Innovation Center for Biomedical Engineering, School of Biological Science and Medical Engineering, School of Engineering Medicine, Beihang University, Beijing 100083, China; Key Laboratory of Biomechanics and Mechanobiology (Beihang University), Ministry of Education, Beijing Advanced Innovation Center for Biomedical Engineering, School of Biological Science and Medical Engineering, School of Engineering Medicine, Beihang University, Beijing 100083, China; State Key Laboratory of Virtual Reality Technology and Systems, Beihang University, Beijing 100083, China; Key Laboratory of Biomechanics and Mechanobiology (Beihang University), Ministry of Education, Beijing Advanced Innovation Center for Biomedical Engineering, School of Biological Science and Medical Engineering, School of Engineering Medicine, Beihang University, Beijing 100083, China; State Key Laboratory of Virtual Reality Technology and Systems, Beihang University, Beijing 100083, China

**Keywords:** poly(glycerol-dodecanoate), *in vitro* degradation, mechanical load, surface erosion, numerical simulation

## Abstract

Poly(glycerol-dodecanoate) (PGD) has aroused increasing attention in biomedical engineering for its degradability, shape memory and rubber-like mechanical properties, giving it potential to fabricate intelligent implants for soft tissues. Adjustable degradation is important for biodegradable implants and is affected by various factors. The mechanical load has been shown to play an important role in regulating polymer degradation *in vivo*. An in-depth investigation of PGD degradation under mechanical load is essential for adjusting its degradation behavior after implantation, further guiding to regulate degradation behavior of soft tissue implants made by PGD. *In vitro* degradation of PGD under different compressive and tensile load has proceeded in this study and describes the relationships by empirical equations. Based on the equations, a continuum damage model is designed to simulate surface erosion degradation of PGD under stress through finite element analysis, which provides a protocol for PGD implants with different geometric structures at varied mechanical conditions and provides solutions for predicting *in vivo* degradation processes, stress distribution during degradation and optimization of the loaded drug release.

## Graphical abstract

**Figure:**
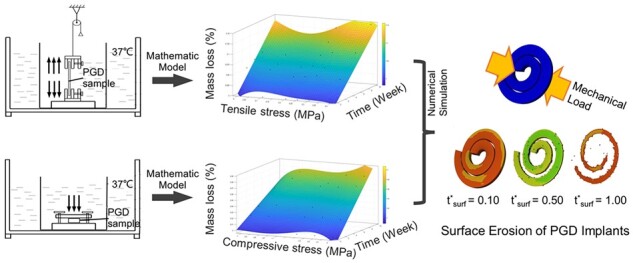

## Introduction

Poly(glycerol-dodecanoate) (PGD) is a promising biodegradable thermosetting polyester that can achieve body temperature-triggered shape memory properties by adjusting its synthesis parameters [1–3]. The glass transition temperature of PGD is between room temperature and body temperature, thus it owns glassy state at room temperature with elastoplasticity, and amorphous state at body temperature with rubber-like properties close to soft tissue [4–6]. These characteristics give PGD the potential to be an intelligent implant material for soft tissues [4]. Degradation property is important for biomedical implants, which is correlated with matching tissue regeneration time, maintaining mechanical support, controlling drug release kinetics etc. [7, 8]. *In vivo* degradation of biodegradable polyester is influenced by multiple external factors, and mechanical loading is one of the key factors after implantation [9, 10]. Soft tissues such as cartilage, intervertebral discs, ligaments and muscles possess a relatively complex mechanical environment, resulting in a non-uniform distribution of stress in the implants, which may lead to non-uniform degradation of the implants and affect its long-term functional maintenance [10].

Mechanical loading has been shown to promote the mechanochemistry of polyesters like PGD and affect activation energy to depolymerize its ester bond, thus significantly changing the mechanical properties and degradation of implant made by the polyester [11, 12]. *In vitro* and *in vivo* degradation studies reveal that polyester can significantly affect the hydrolysis under static or dynamic mechanical loading compared to non-mechanical loading conditions [13–16]. Degradation of poly(glycolic acid) (PGA) sutures is accelerated under tensile stress, it breaks at 17 days after implantation, while PGA suture without tensile stress breaks until 25 days after implantation [17]; degradation of poly(l-lactide) (PLLA) under cyclic mechanical loading is faster significantly than that not subjected to mechanical loading [18]. In addition, mechanical environments of the implants made by polyester with variable structures are more complex, which makes it difficult to control degradation process after implantation [19, 20]. For PGD implants, previous studies have revealed surface erosion mechanism and linear mass loss with both *in vitro* and *in vivo* experiments, but have not investigated its degradation under mechanical load [1, 5]. An in-depth investigation of PGD degradation under mechanical load is essential for adjusting its degradation behavior after implantation, further guiding to regulate degradation behavior of soft tissue implants made by PGD.

Numerical simulation has been widely used in degradation simulation of biomaterials and bioimplants for its high efficiency and low cost compared with experimental tests [21]. For biomaterials degraded with surface erosion mechanism such as PGD, a continuous damage model with erosion layers is introduced to simulate its degradation process based on the characteristic of gradual erosion from the surface to the inside [22, 23]. Our previous study employs the model to simulate degradation process of surface erosion material under mechanical loading, and approves good correlation with our *in vivo* degradation [20, 24, 25]. We suppose to obtain degradation property of PGD under different mechanical load by experiments and describe the relationships by empirical equations, then simulate PGD implants degradation under mechanical loading by finite element analysis (FEA) program with the continuous damage model and the empirical equations.

In this study, *in vitro* degradation experiments are performed on PGD specimens under varied tensile and compressive loading using a self-made loading device, and the varying mass loss, morphology and mechanical properties of PGD specimens are evaluated. Empirical equations are developed to quantitatively describe the relationship between mass loss and stress intensity during the degradation experiments. Based on the above equations, a continuum damage model is designed to simulate surface erosion degradation of PGD under stress, enables the degradation simulation of PGD implants after implantation, and facilitates the optimization of implant structures and prediction of degradation duration and drug release kinetics according to clinical needs.

## Experimental section

### Synthesis PGD specimens

**Figure 1.**
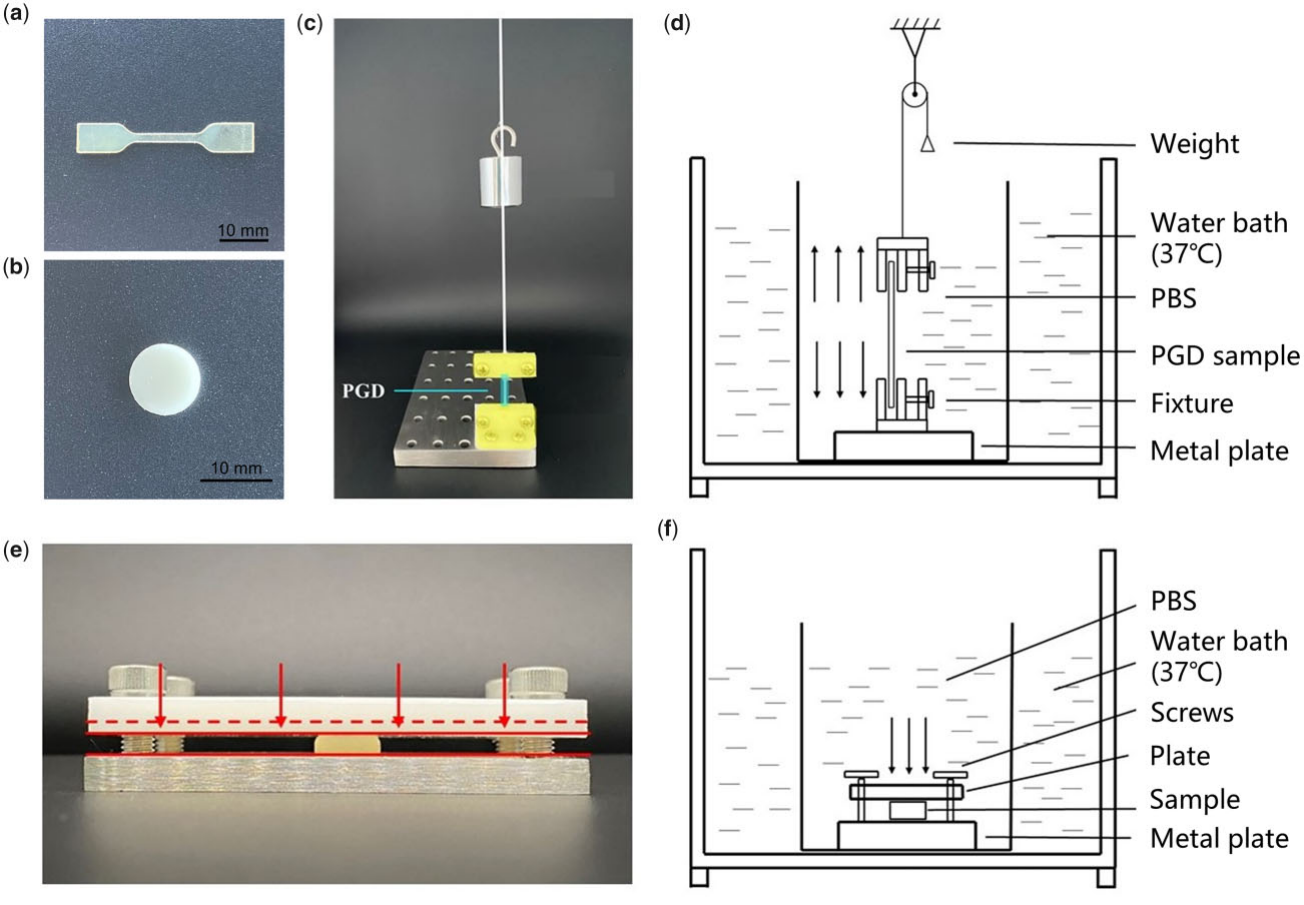
PGD specimens and the self-made mechanical load devices. (**a**) Tensile specimen made by PGD. (**b**) Compressive specimen made by PGD. (**c**) Tensile load device used for PGD specimens’ degradation under tensile stress load. The highlighted box indicates clamps fixed PGD specimen. (**d**) Schematic of PGD degradation under tensile stress load. (**e**) Compressive load device used for PGD specimens’ degradation under compressive stress load. (**f**) Schematic of PGD degradation under compressive stress load.

Glycerin and dodecanedioic acid with equal molar ratios were mixed at 120°C under nitrogen flow for 24 h. The product further reacted under a −0.08 MPa vacuum environment at 120°C for another 24 h to obtain the PGD prepolymer (Supplementary Fig. S1). PGD sheets with 1 and 3 mm thickness were synthesized by reacting its prepolymer under vacuum condition at 120°C for 120 h as described previously [2]. Tensile specimens were laser cut from the 1 mm thickness PGD sheets following ISO527-5B standard (Fig. 1a). Compress specimens with 10 mm diameter and 3 mm thickness were also fabricated by the 3 mm thickness PGD sheets (Fig. 1b). Material density of PGD was measured after its synthesis using Archimedes drainage method, and calculated as the following equation shows: where *m* represents the weight of PGD specimens (*n* = 5) measured by precision balance (XPR226, Mettler Toledo), *v*_1_ represents volume of water in pycnometer, *v*_2_ represents volume of water in pycnometer with PGD specimens.


(1) $$\rho =\frac{m}{v2-v1},$$

### Thermodynamic properties of PGD

Thermodynamic properties of PGD were evaluated using differential scanning calorimetry (DSC8000, PerkinElmer). PGD specimens (*n* = 3) were placed in an aluminum crucible and subsequently preheated from 25°C to 90°C at a rate of 10°C/min for 3 min to eliminate thermal history. The specimens were cooled to −30°C at a rate of 10°C/min and then heated to 80°C at a rate of 10°C/min. Variation in heat flow during the period was recorded, and melting crystals and crystallization processes were analyzed.

### Mechanical properties of PGD

Mechanical properties of PGD at 37°C were evaluated using a mechanical testing machine with temperature controller (IPBF-300S, CARE). Tensile specimens (*n* = 3) were stretched at a rate of 10 mm/min until broke. Poisson’s ratio of PGD was measured simultaneously using a video extensometer, which was calculated as follows: where ε_*x*_ is the transverse strain of the specimen and ε_*y*_ is the longitudinal strain of the specimen. In the uniaxial compression test, the compress specimen of PGD (*n* = 3) was compressed at a rate of 1 mm/min and stopped when the compression strain reaches 66.7% of its original state. The degraded PGD specimens (*n* = 3) were also tested separately for tensile and compressive mechanical properties according to the above-mentioned methods. The crosslink density (*n*, mol/m^3^) of PGD was calculated from the following formula, as previously mentioned [2]: where *R* represents the universal gas constant (8.3114 J/mol K), *T* represents the absolute temperature during testing (K) and *E* represents Young’s modulus of PGD (*E*, Pa).


(2) $$\mu =\frac{\varepsilon_{x}}{\varepsilon_{y}},$$

(3) $$n=\frac{E}{3RT},$$

### *In vitro* degradation of PGD specimens with external mechanical load

Tensile and compressive loading devices used for the *in vitro* degradation experiments were shown in Fig. 1c and d and Fig. 1e and f, respectively. The tensile loading device was worked by clamping the two ends of the PGD tensile specimen with upper and lower clamps, where the lower clamp was fixed on a stainless-steel base and the upper clamp was connected to weights with a fishing line. Tensile stress loads of 0.1, 0.2, 0.25, 0.3, 0.35 and 0.4 MPa were applied to each group of PGD specimens by varying the weights mass. The compressive loading device was used to compress PGD specimens to different displacements by its upper clamp. Each group was subjected to a compression load of 0.2, 0.4, 0.6, 0.8 and 1.0 MPa. Control group with no load was set up for PGD specimens on the tension and compression devices, respectively. Experimental groups (*n* = 12) were degraded in PBS at 37°C, and collected specimens at 2, 4, 6, 8 and 10 weeks, respectively. Six of the 12 specimens in each group were used to measure mechanical property, the other was used to evaluate mass loss and micromorphology after degradation.

### Mass loss of PGD specimens after degradation

The degraded PGD specimens were rinsed with pure water to remove the phosphate on their surface, and then weighed after completely dried in vacuum desiccator for more than 5 days. Mass loss of the specimens was calculated as following: where *m*_0_ represents the specimen mass before degradation, *m* represents the specimen mass after degradation.


(4) $$\text{mass loss}\;(\%)=\frac{m_{0}-m}{m_{0}}\times 100\%,$$

### Morphology of PGD specimens after degradation

Surface and cross-sectional morphology of PGD specimens were observed using scanning electron microscopy (SEM) (Quanta 250FEG, FEI). The fully dried PGD specimens were cut from the surface and cross-section using a sharp microtome blade. Platinum with 200 nm thickness was uniformly sprayed onto the specimen surface to promote its electrical conductivity. PGD specimens were placed in SEM and evacuated, followed by observation of the morphology using a secondary electron detector at 3000× field.

### Degradation simulation of PGD vortex implant under mechanical load

A 3D model of PGD implant with vortex structure (∼6 mm diameter, 0.5 mm thickness) was designed by CAD software. The 3D model was imported into Hypermesh software for hexahedral element meshing, and the mesh size was set to 0.05 mm according to previous mentioned effective erosion thickness of polymer [26, 27], finally to obtain a finite element model of vortex implant with a total number of 60 000 elements. Material properties of the vortex implant were chosen as that of PGD under compressive stress, since the implant was under compressive load after implantation. The material was set to describe PGD, as shown in Table 1. All the nodes on the bottom surface of implant model were defined, where the displacements in *x* and *y* directions were not constrained and the remaining four freedom degrees were set as zero. A 0.01 MPa compressive load was then applied to the upper surface of the implant model.

**Table 1.** Mechanical properties of PGD at 37°C

Material properties	PGD tensile specimens at 37°C	PGD compressive specimens at 37°C
Material density	1.31 g/cm^3^	
Poisson’s ratio	0.47	
Young’s modulus	2.30 MPa	0.85 MPa
Yield stress	0.14 MPa	0.34 MPa
Fracture stress	0.88 MPa	N/A
Fracture strain	49.37%	N/A

**Figure 2.**
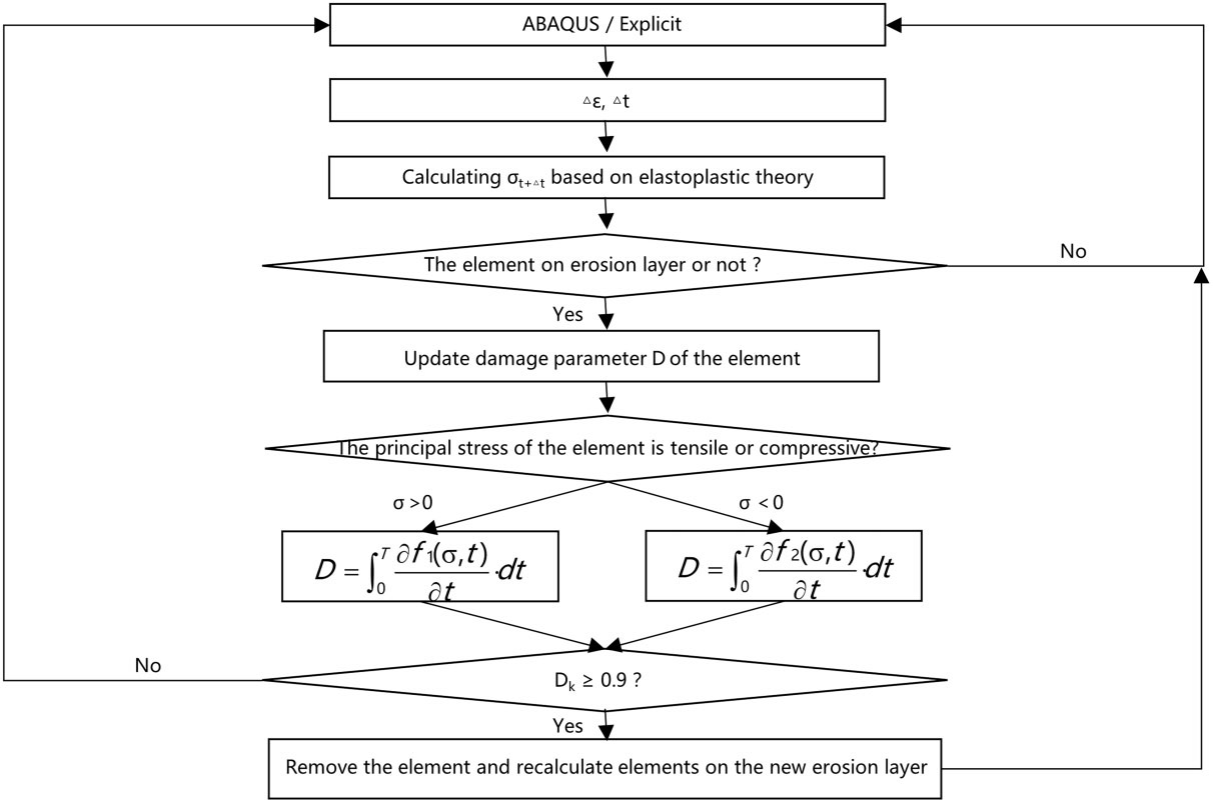
Flowchart for the surface erosion degradation model VUMAT.

Surface erosion of each element in our FEA simulation was assumed to be the stress erosion process, as shown in Fig. 2. The degradation of the elements was described by a continuum damage model and employed a scalar damage parameter, *D*, which was monotonically increasing from 0 to 1 during erosion to determine the changes of mechanics integrality from undamaged elements (*D* = 0) to completely damaged elements (*D* = 1), and described as follow: where *D* is related to the element damage resulting from stress induced erosion. *f*_1_(*σ, t*) is empirical equation that described mass loss of PGD specimen under tensile stress load and conclude from experiments. *f*_2_(*σ, t*) is empirical equation that described mass loss of PGD specimen under compressive stress load. *σ* is the principal stress of element which is greater than zero means the element under tensile stress load, and less than zero means the element is under compressive stress load.


(5) $$D=\int_{0}^{T}\frac{\partial f_{1}(\sigma ,t)}{\partial t}\cdot d\mathrm{t,}\;\mathrm{when}\;\sigma >0$$

(6) $$D=\int_{0}^{T}\frac{\partial f_{2}(\sigma ,t)}{\partial t}\cdot d\mathrm{t,}\;\mathrm{when}\;\sigma \leq 0$$

The VUMAT code was employed to the PGD implant model to update the stress of the elements, receiving a strain and time increment from Abaqus solver at the beginning of an analysis increment, and returned the resulting scalar damage parameter at the end of the analysis increment. The surface erosion process was considered on an element-by-element removing, and only occurred for the elements on exposed erosion layer of the implant model. The VUMAT received the element connectivity map by reading an input file, then the elements on the erosion layer were determined and finally removed when its *D* reached 0.9. The elements around the removed element were exposed and updated to the refreshed erosion layer.

## Results

### Physicochemical characteristics of PGD polymer

**Figure 3.**
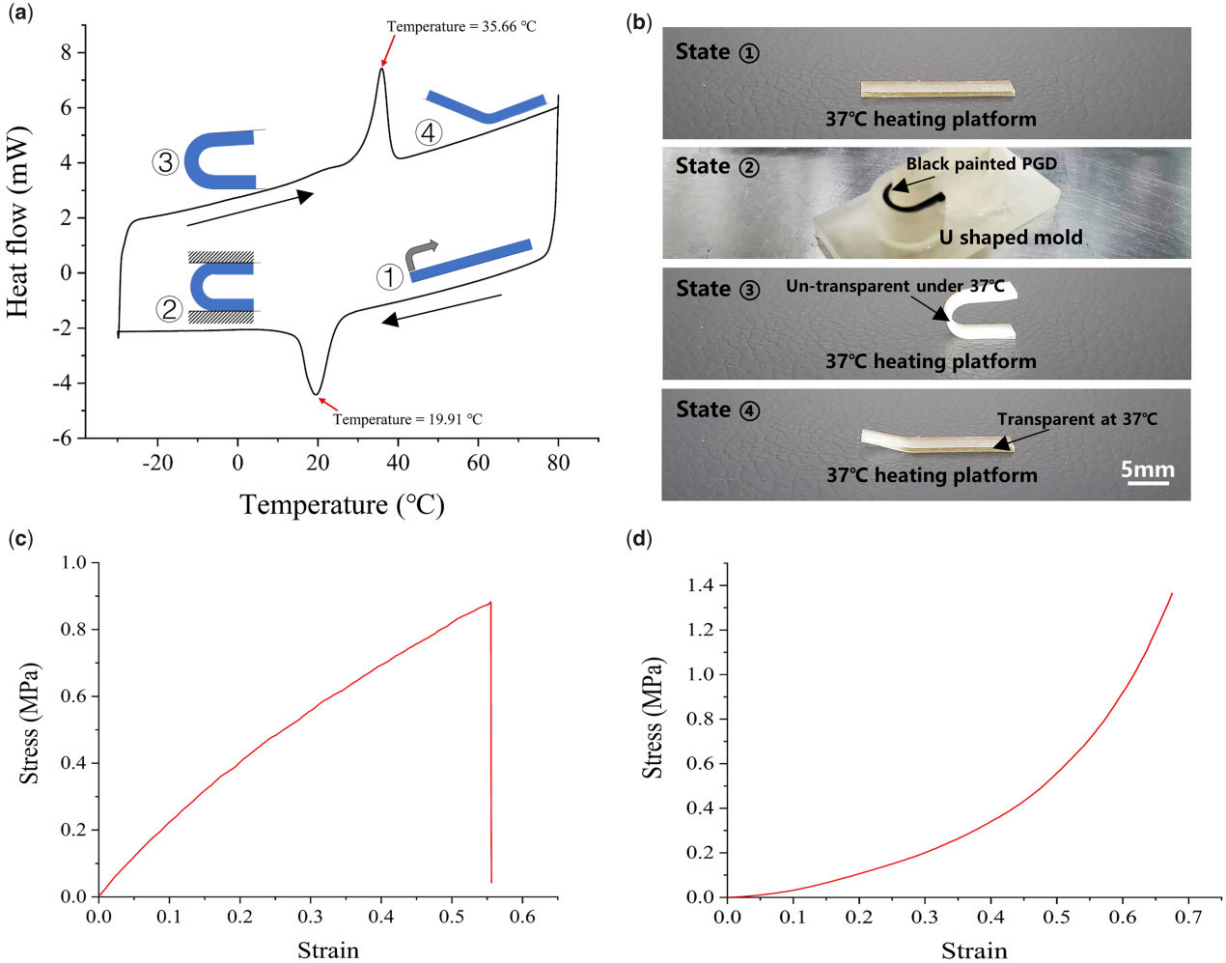
Shape memory and mechanical characteristics of PGD polymer. (**a**) Thermodynamic properties of PGD used for degradation. (**b**) Shape program and shape recover process of PGD polymer between 37°C and 20°C. (**c**) Typical stress–strain curve of PGD under tensile load at 37°C. (**d**) Typical compressive stress–strain curve of PGD under compressive load at 37°C.

Thermal, shape memory and mechanical properties of PGD polymer were investigated before degradation. Heat flow-temperature curves of PGD were measured by DSC, as shown in Fig. 3a. A typical semi-crystalline polymer was detected via an exothermic peak during cooling and an endothermic peak during heating, which was similar with previous reported PGD characteristic [1–3]. The exothermic and endothermic peak temperatures of PGD polymer in our study were 20.56 ± 0.79°C and 34.67 ± 0.75°C. The crystals in the polymer chains were formed or melted when the temperature of PGD was lower or higher than the exothermic or endothermic peak temperatures, respectively [3, 28]. This phenomenon gave PGD amorphous state with rubber-like property at 37°C and glassy state with plasticity at 20°C [29]. The phase change property between 37°C and 20°C could be used to program the shape of PGD polymer. As shown in Fig. 3b, the shape of PGD polymer was programed by a U-shaped mold at body temperature and kept the U shape when the polymer temperature cooled to room temperature. The U-shaped PGD was returned to its original shape when the polymer temperature back to body temperature again. Shape memory ability of PGD was able to make body temperature-triggered minimal invasive implants [5]. As shown in Fig. 3c and d, mechanical properties of PGD specimens at body temperature were measured via tensile and compressive conditions. Young’s modulus for the tension experiment was 2.3 MPa, while Young’s modulus for the compression experiment was 0.85 MPa. The Poisson’s ratio of PGD was 0.47, and the material density was 1.31 g/cm^3^.

### *In vitro* degradation of PGD under tensile and compressive mechanical load

**Figure 4.**
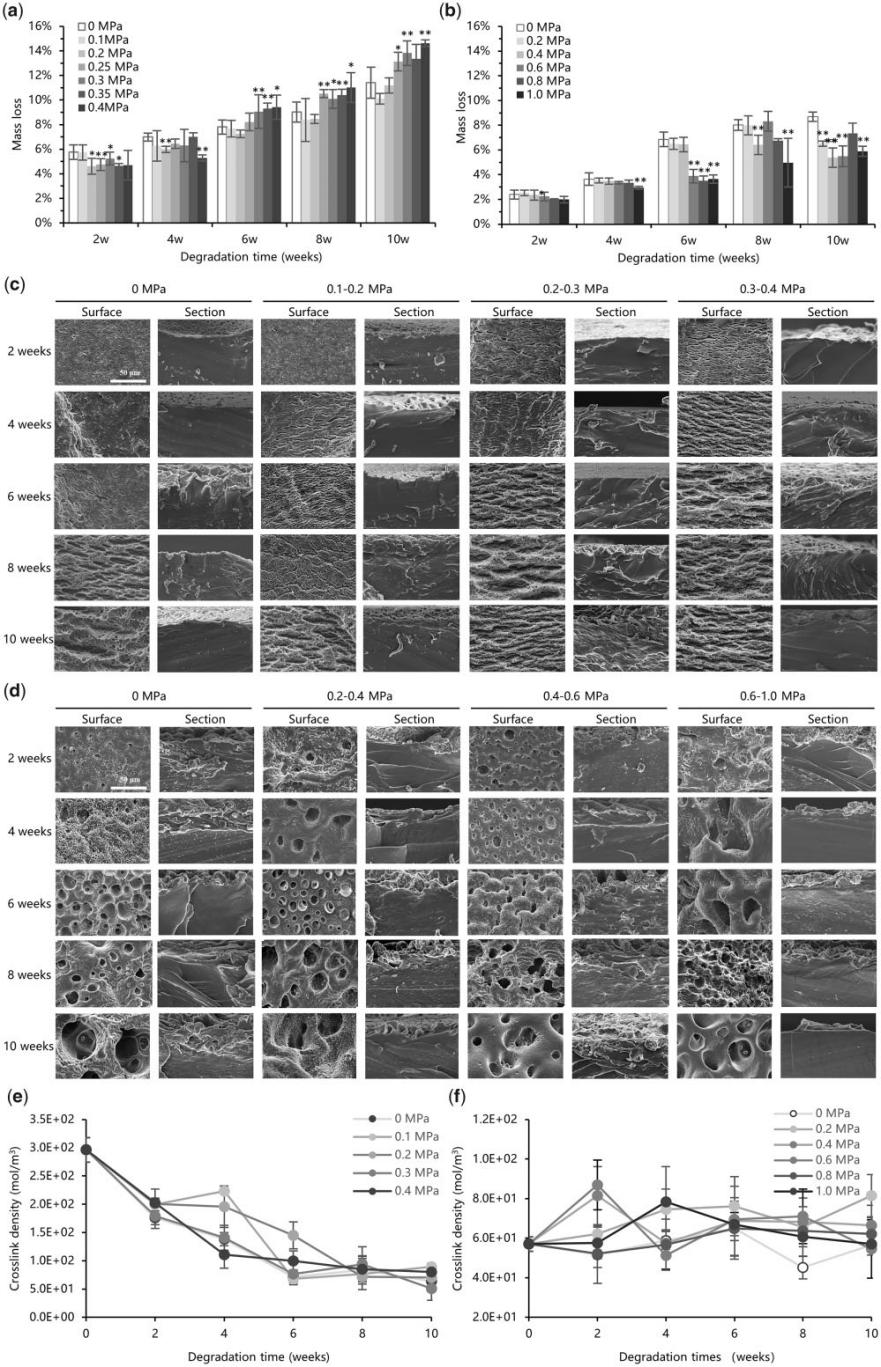
*In vitro* degradation of PGD specimens under mechanical load. Mass loss of PGD specimens under tensile stress load (**a**) and compressive stress load (**b**). Stars in the histogram indicate significant differences between the two groups determined by independent sample *t* testing (*P* < 0.05). Surface and cross-section morphology of PGD specimens under tensile stress load (**c**) and compressive stress load (**d**). SEM parameters: HV 20.00 kV, mag ×3000, mod SE, det ETD. Crosslink density of PGD specimens under tensile stress load (**e**) and compressive stress load (**f**).

Mass loss of PGD specimens under tensile and compressive load during degradation was shown in Fig. 4a and b. It showed that mass loss of PGD tends to increase linearly with time whether in compression, tension or without mechanical load. Degradation of PGD under tensile load tended to be faster than that without mechanical load (Fig. 4a). Mass loss of each tensile load group was generally around 5–6% at 2 and 4 weeks after degradation, which was not different significantly from the control group. Mass loss of the groups with higher tensile stress (0.35–0.4 MPa) started to be degraded faster than that of the control group from 6 weeks, while the groups with lower tensile stress (0.1–0.3 MPa) had similar mass loss with the control group. This trend continued consistently until 10 weeks, when Groups 0.3 and 0.4 MPa lost ∼14% of their initial mass, while Groups 0.1 and 0.2 MPa and the control group lost ∼10%. Degradation of PGD under compressive load tended to be slower than that without mechanical load (Fig. 4b). Mass loss in each group was no different from that of the control group at 2 weeks after degradation, which was ∼2% of their initial mass. Group 1.0 MPa began to show a slower trend than the control from 4 weeks. Mass loss of the groups with higher compressive stress (0.6–1.0 MPa) was ∼4% at 6 weeks, and the degradation was significantly slower than that of the control group and the groups with lower compressive stress which was ∼6%. Mass loss in each compressive stress group was significantly lower than that of the control group at 10 weeks.

Surface and cross-section morphology of PGD specimens after degradation under mechanical load were shown in Fig. 4c and d. Surface of PGD under tensile and compressive load presented different morphological characteristics. Corrugated erosion along the tensile direction was distributed on degraded PGD specimens under tensile stress, while round-like erosion showed on degraded PGD specimens under compressive stress. Morphology of groups under tensile stress was shown in Fig. 4c, it showed that macro corrugated erosion along the tensile direction appears on PGD surface at first time, and then increasing corrugated erosion was formed and gradually became granulation with the degradation progresses. No significant changes were observed on surfaces of control group and Groups 0.1–0.2 MPa at 2 weeks after degradation, while erosion pores started to appear in Groups 0.2–0.4 MPa. Control group and Groups 0.1–0.2 MPa started to show surface erosion after 6 weeks. of degradation. Surface erosion of Groups 0.2–0.4 MPa became severe at 8 and 10 weeks, and the number of erosion pores increased. Morphology of groups under compressive stress was shown in Fig. 4d. Erosion pores of PGD under compressive load showed nearly circular, which was different from that of under tensile load. The number of erosion pores on PGD gradual increases with degradation process, but there was no significant difference among each group. Cross-section of PGD did not show erosion pores no matter under tensile or compressive load, indicating that the degradation occurred only on the surface of PGD and did not extend to its interior.

Young’s modulus of PGD specimen after degradation was measured as shown in Tables 2 and 3, and their crosslink densities were counted to reflect the variation of network molecules in the thermoset polymer, as shown in Fig. 4e and f. For PGD degradation under tensile load, crosslink density of each group gradually decreased with degradation time. At 10 weeks, crosslink density of PGD specimens (Groups 0, 0.1, 0.2, 0.25, 0.3, 0.35 and 0.4 MPa) was 78.6%, 70.0%, 76.3%, 72.1%, 82.9% and 73.0% lower than their initial states before degradation. For PGD degradation under compressive load, crosslink density of each group maintained their initial states during 10 weeks degradation.

**Table 2.** Young’s modulus of tensile specimens after degradation

Groups	Time				
	2 weeks (MPa)	4 weeks (MPa)	6 weeks (MPa)	8 weeks (MPa)	10 weeks (MPa)
Tens. 0.0	1.36 ± 0.15	1.07 ± 0.09	0.55 ± 0.08	0.64 ± 0.19	0.49 ± 0.08
Tens. 0.1	1.54 ± 0.09	1.73 ± 0.02	0.53 ± 0.08	0.59 ± 0.21	0.69 ± 0.05
Tens. 0.2	1.55 ± 0.20	1.51 ± 0.28	1.12 ± 0.18	0.56 ± 0.05	0.55 ± 0.09
Tens. 0.3	1.39 ± 0.12	1.09 ± 0.17	0.59 ± 0.05	0.73 ± 0.24	0.39 ± 0.16
Tens. 0.4	1.56 ± 0.05	0.86 ± 0.19	0.77 ± 0.14	0.66 ± 0.17	0.62 ± 0.11

**Table 3.** Young’s modulus of compressive specimens after degradation

Groups	Time				
	2 weeks (MPa)	4 weeks (MPa)	6 weeks (MPa)	8 weeks (MPa)	10 weeks (MPa)
Comp. 0.0	0.40 ± 0.11	0.46 ± 0.03	0.50 ± 0.11	0.35 ± 0.04	0.44 ± 0.01
Comp. 0.2	0.48 ± 0.08	0.58 ± 0.08	0.59 ± 0.12	0.51 ± 0.11	0.63 ± 0.08
Comp. 0.4	0.63 ± 0.11	0.44 ± 0.05	0.53 ± 0.13	0.53 ± 0.02	0.51 ± 0.08
Comp. 0.6	0.67 ± 0.10	0.40 ± 0.05	0.54 ± 0.03	0.55 ± 0.11	0.42 ± 0.11
Comp. 0.8	0.40 ± 0.05	0.44 ± 0.10	0.50 ± 0.12	0.49 ± 0.06	0.48 ± 0.06
Comp. 1.0	0.45 ± 0.06	0.61 ± 0.14	0.52 ± 0.16	0.47 ± 0.12	0.44 ± 0.04

### PGD degradation equation under tensile and compressive load

**Figure 5.**
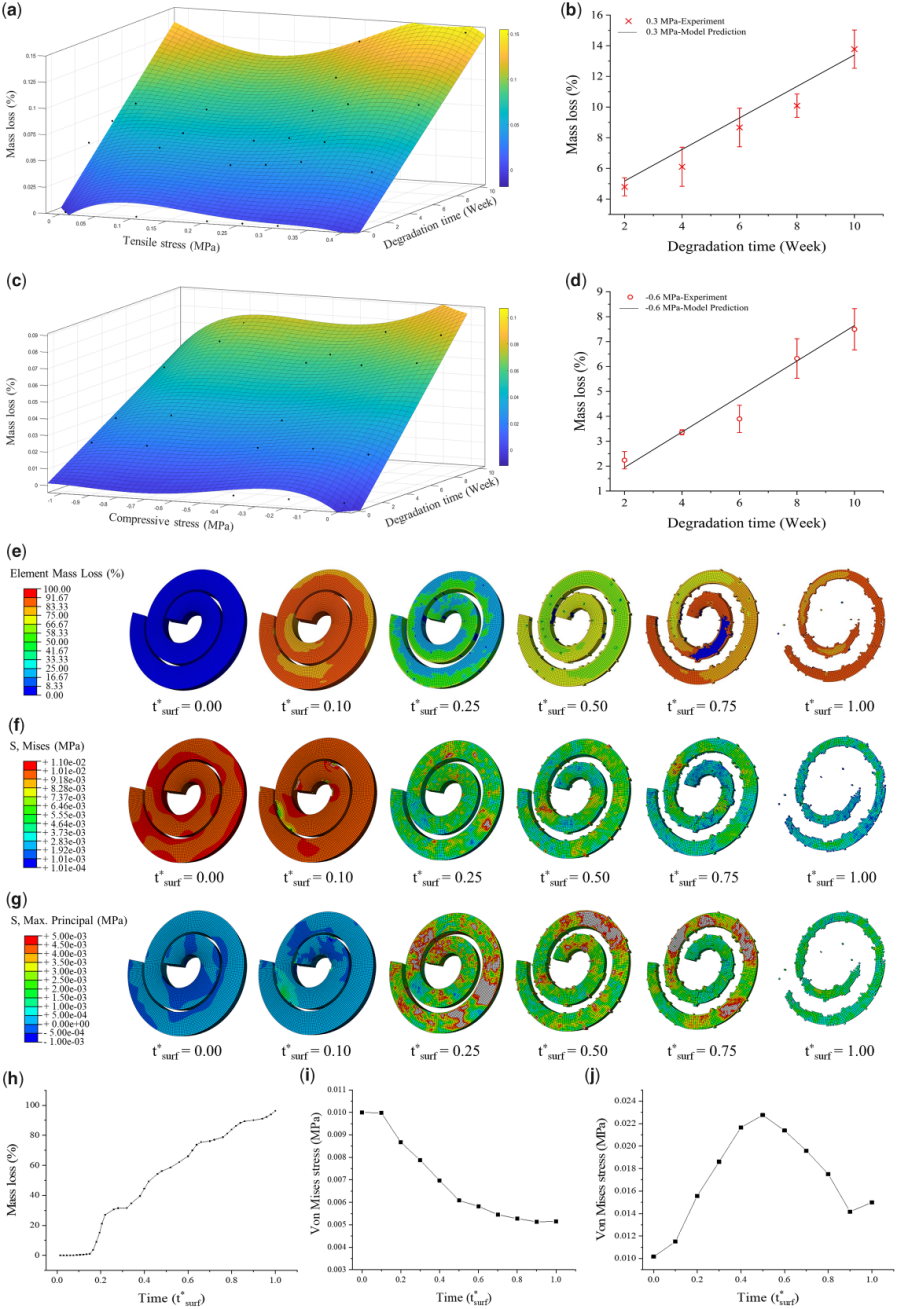
Degradation simulation of PGD implant under mechanical load. (**a**) Surface fitting of PGD degradation under tensile stress load. (**b**) Mass loss predicted by empirical Equation (7) compared with experimental dataset (*n* = 6). (**c**) Surface fitting of PGD degradation under compressive stress load. (**d**) Mass loss predicted by empirical Equation (8) compared with experimental dataset (*n* = 6). (**e**) Element mass loss of PGD implant during surface erosion simulation based on our FEA program. (**f**) Stress distribution of PGD implant based on our FEA program. (**g**) Maximum stress distribution of PGD implant based on our FEA program. Simulated mass loss (**h**), mises stress (**i**) and maximum stress (**j**) of PGD implant during degradation at mechanical load condition.

Six groups of experimental data (0, 0.1, 0.2, 0.25, 0.35 and 0.4 MPa) were chosen as the base data to fit the mass loss under tensile stress load. The empirical equation was obtained by fitting above-mentioned experimental data through MATLAB, and described relationship among mass loss, stress intensity and degradation time as Equation (7) and plotted as shown in Fig. 5a: where *f*_1_(σ, *t*) is the mass loss of PGD under tensile stress, *σ* is the tensile stress, *t* is the degradation time and *a*_1_ ∼ *j*_1_ are constants. The results of surface-fitting experimental data with Equation (6) were given in Table 4. Determination coefficient *R*^2^ of the fitted equation was 0.955.


(7) $$f_{1}(\sigma ,t)=a_{1}\sigma +b_{1}t+c_{1}\sigma^{2}+d_{1}\sigma t+e_{1}\sigma^{3}+f_{1}\sigma^{2}t+g_{1}\sigma^{4}+h_{1}\sigma^{3}t+i_{1}\sigma^{5}+j_{1}\sigma^{4}t,$$

**Table 4.** Surface-fitting results of mass loss and tensile stress during degradation

Constants		95% confident bounds
*a*_1_	138.90	(−5.65, 283.50)
*b*_1_	0.829	(0.570, 1.088)
*c*_1_	−2516	(−5040.00, 7.61)
*d*_1_	−12.73	(−24.93, −0.53)
*e*_1_	14 630	(−1060, 30 330)
*f*_1_	139.60	(−2.75, 281.90)
*g*_1_	−34 750	(−75650, 6148)
*h*_1_	−482.10	(−1037.00, 73.13)
*i*_1_	28 980	(−9190, 67 160)
*j*_1_	551.9	(−138.2, 1242.0)

Five groups of experimental data (0, 0.2, 0.4, 0.8 and 1.0 MPa) were chosen as base data to fit the mass loss under compressive load. The relationship among mass loss, stress intensity and degradation time were described as Equation (8) and plotted as shown in Fig. 5c: where *f*_2_(σ, *t*) is mass loss under compressive stress, *σ* is the compressive stress, *t* is the degradation time and *a*_2_ ∼ *h*_2_ are constants. The results of surface-fitting experimental data with Equation (7) were given in Table 5. Determination coefficient *R*^2^ of the fitted equation was 0.937.


(8) $$f_{2}(\sigma ,t)=a_{2}\sigma +b_{2}t+c_{2}\sigma^{2}+d_{2}\sigma t+e_{2}\sigma^{3}+f_{2}\sigma^{2}t+g_{2}\sigma^{4}+h_{2}\sigma^{3}t,$$

**Table 5.** Surface-fitting results of mass loss and compressive stress during degradation

Constants		95% confident bounds
*a*_2_	−8.04	(−27.43, 11.33)
*b*_2_	0.870	(0.640, 1.099)
*c*_2_	−33.20	(−111.30, 44.91)
*d*_2_	0.900	(−1.335, 3.134)
*e*_2_	−42.46	(−154.30, 69.42)
*f*_2_	1.829	(−3.941, 7.599)
*g*_2_	−17.19	(−70.34, 35.97)
*h*_2_	1.277	(−2.643, 5.197)

Degradation equations predicting the mass loss under tensile and compressive stress were compared with the experimental datasets, respectively. As shown in Fig. 5b and d. The equation predictions for 0.3 MPa tensile load and 0.6 MPa compressive load agreed well with experimental data.

### Degradation simulation of implants made by PGD

As shown in Fig. 5e, the vortex implant made by PGD degraded like typical surface erosion polymer in the FEA degradation simulation, which showed an orderly erosion from surface to inside. The implant started to lose surface elements at t^*^_surf_ = 0.10, and its structure fractured at t^*^_surf_ = 1.000. Mises’s stress and maximum stress distribution of the implant varied as implant elements deleted during degradation, as shown in Fig. 5f and g. The simulated mass loss of the PGD implant showed linearly trend as previous *in vitro* and *in vivo* results (Fig. 5h). Mises’s stress on the PGD implants gradually decreases and reaches to 50% of its initial state at t^*^_surf_ = 0.6 as shown in Fig. 5i. Maximum stress on the PGD implants showed an increasing trend from t^*^_surf_ = 0 to t^*^_surf_ = ∼0.5, following a decreasing trend from t^*^_surf_ = ∼0.5 to t^*^_surf_ = 1. The area that suffered greater tensile stress tended to degrade faster, while the area that suffered greater compressive stress tended to degrade slower based on our degradation equations. It helps to determine where the maximum stress value occurs during implant degradation and guides the subsequent optimization of the implant structure.

## Discussion

PGD degradation is affected by mechanical environment it located, which causes inconsistent degradation in different parts of implants made by PGD after implantation. Adverse degradation *in vivo* may lead to implant failure, so it is essential to have insight into the relationship between PGD degradation and mechanical load during implantation. In this study, we investigated PGD degradation under varied tensile or compressive stresses load, and used polynomial equation to quantitatively describe the relationship between mass loss and stress intensity during degradation. Based on the above equations, a continuum damage model was designed to simulate surface erosion degradation of PGD under stress, which provided a protocol for PGD implants with different geometric structures at varied mechanical conditions.

PGD degradation mechanism exhibited typical surface erosion characteristics both with or without stress load. Previous studies had confirmed through *in vitro* and *in vivo* experiments that PGD specimens without stress load have the following degradation characteristics: gradually decreased specimen dimension, erosion pores on the outer surface but not inside, low water absorption and linear mass loss [27, 30, 31]. These characteristics were similar to those of previous mentioned surface erosion polymer and clearly different from the polymer with bulk erosion degradation mechanism [31, 32]. In our study, PGD specimens under varied tensile or compressive load shared similar morphology and mass loss with above-mentioned characteristics, indicating mechanical load did not change surface erosion mechanism of PGD.

We set up several tensile load groups from 0.1 to 1.0 MPa, in which the 1.0 MPa group fractured within 1 week after degradation, while most of the 0.5 MPa group specimens fractured at 8 weeks after degradation. PGD groups under stress load lower than 0.4 MPa remained intact dimensions to measure material properties after 10 weeks degradation. The tensile stress load acting on PGD specimens accelerated its degradation, and the mechanochemical effect was related to the phenomenon. According to mechanochemistry, tensile stress applied to PGD increased its activation energy for promoting the ester bond breaking efficiency during degradation and accelerated PGD hydrolysis process [11, 12]. The same results were also observed from other biopolymers, such as poly(l-lactide-coglycolide) (PLGA) and PLLA [13, 18, 33]. Unlike the degradation under no stress load, the erosion pores of PGD under tensile stress load showed a long strip distribution along the stress direction. Expansion of the oriented erosion pores was easy under tensile stress, furtherly increasing the exposure area between PGD specimens and the liquid, thus accelerating the hydrolysis process. As for the relationship between stress load and degradation rate, we found that the erosion process of Groups 0.3–0.4 MPa was significantly faster than those of Group 0.1–0.3 MPa and the control group from sixth week after degradation; tensile stress mediated accelerated degradation obviously functioned for a certain time and threshold value; this result was consistent with the mechanochemical theory and surface morphology observations.

For loading ranges during compressive degradation, 0.2–1.0 MPa was selected to match young’s modulus of soft tissue in body, such as tendon, skin, intervertebral disc and cartilage (Supplementary Fig. S2) [34]. The compressive stress load acting on PGD specimens slowed down its degradation. The mass loss of Group 1.0 MPa showed slower than the other groups and control group from second week after degradation, and those of the other groups showed the same trend at 8–10 weeks. Erosion pores of PGD specimens under compressive stress load were rounded and did not change with increasing stress load, which was obviously different from those of PGD specimens under tensile stress load. The difference between groups under compressive and tensile stress load was also reflected in the network molecule changes during degradation, where the crosslink density of compressive load groups showed fluctuant during the 10 weeks degradation. Linearly fitting the crosslink density—degradation time of each group was found that their crosslink densities did not change significantly within 10 weeks. These phenomena were not only shown for PGD but other biodegradable materials under compressive stress, showing a different factor affected degradation of PGD specimens under compressive stress load [25, 35, 36]. According to mechanochemical theory, increased stress load should lead to an accelerating degradation rate similar to those of groups with tensile stress load. Physical effects under compressive stress load were considered as primary reason affected PGD degradation under compressive stress load other than mechanochemical effects, because the compression caused collapsed morphology of both macro and micro scale, restricted expansion of contact area that hydrolysis happened and diffusion of infiltrated liquid, thus reduced the efficiency of PGD specimen degradation [37].

The difference in the pore morphology of the control groups was related to the varied specimen shape and degradation conditions during tensile/compression degradation experiments. Control group of the tensile degradation was dog bone-shaped and stretched naturally in 37°C PBS solution during degradation. Control group of the compressive degradation was cylinder shaped and covered by the plate from top and bottom in 37°C PBS solution during degradation. The contact surface with PBS solution was different between the two control groups, which led to the varied surface morphology. Besides, convection in 37°C PBS solution might also affect micro-morphology of the polymer specimen during experiments (Supplementary Fig. S3), which was similar to previous reported polymer disc [38]. To evaluate the degradation between the control groups of tensile and compressive experiment, it was necessary to eliminate surface area factor among them. Mass loss per unit area was employed to eliminate surface area factor between the control groups, the equation was listed as following:


(9) $$\text{Mass loss per unit area}\;\left(\text{mg}/{\text{mm}}^{2}\right)=\frac{m_{0}-m}{S},$$

where *m*_0_ (mg) represented the mass of PGD specimen before degradation; *m* represented the mass of PGD specimen after degradation; *S* (mm^2^) represented the surface area of PGD specimens after degradation. The results were shown in Supplementary Fig. S4, indicating a similar degradation of the control groups without stress load during the 10 weeks experiment. Therefore, we believed that mass loss results of tensile experiments and compressive experiments (Fig. 4a and b) were comparable, and reasonable to adopt in the FEA simulation.

Based on above-mentioned PGD degradation results, empirical equations [Equations (7) and (8)] were used to describe the relationship among PGD specimen mass—stress intensity—degradation time under tensile and compressive stress load, respectively. The predicted mass loss fitted well with the experimental results from 0.3 MPa tensile load group and 0.6 MPa compressive group, and the determination coefficient *R*^2^ are 0.955 and 0.937 (Fig. 5b and d). It showed that the empirical equation could well predict the PGD degradation under tensile or compressive stress.

A continuum damage model with erosion layer was introduced in our study to characterize surface erosion mechanism of PGD degradation based on our previous works [9, 24, 25]. Degradation simulation of PGD implants under mechanical loading was realized by combining stress analysis of elements in the erosion layer and mass loss calculation by empirical equations. We simulated degradation process of PGD implant under compressive stress loading, and the implant achieved a layer-by-layer erosion degradation just like previously reported *in vivo* degradation of polymer with surface erosion mechanism [1, 5]. Analyzing the Misses stress and maximum stress distribution of the vortex PGD implant also reflected the weak points of the implant structure, which was beneficial for optimal designing its structure before implantation. In addition, our simulation program could be extended to simulate the degradation of other structural PGD implants under stress loading conditions *in vivo*, providing solutions for predicting *in vivo* degradation processes, stress distribution during degradation and optimization of the loaded drug release.

## Conclusion

The loading devices were made to explore PGD degradation under tensile and compressive load, confirming the accelerated degradation of PGD under tensile load and the suppressive degradation of PGD under compressive load. Empirical equations were obtained to describe mass loss of PGD under mechanical load, and the calculated PGD degradation results under tensile or compressive load showed a high correlation with the experimental data. A continuum damage model was designed to simulate surface erosion degradation of PGD under mechanical load, which gave a protocol for PGD implants with different geometric structures at varied mechanical conditions. There were still some limitations in this study that need to fulfill in further studies, such as an improved continuum damage simulation that concerning exposed surface area of each element, long-term degradation data under mechanical load *in vitro* or *in vivo*, the polymer constitutive model in the simulation presenting shape memory properties of PGD implants between body and room temperature etc.

## Supplementary Material

rbad050_Supplementary_Data
