# Supplementary material for: Relationship between mechanical load and surface erosion degradation of a shape memory elastomer poly(glycerol-dodecanoate) for soft tissue implant
Source: Regen Biomater. 2023 May 10;10:rbad050. doi: 10.1093/rb/rbad050 (PMC10219789; doi:10.1093/rb/rbad050)
Supplement: rbad050_Supplementary_Data [file rbad050_supplementary_data.docx]

Reviewer: 1
Comments to the Author
Dear Authors,
After careful review of the manuscript, I’d like to provide following report:
The authors present an in-depth investigation of poly(glycerol-dodecanoate) degradation under mechanical load after implantation. They outlined the degradation simulation of biomaterials and several different mechanical loads that were reported to affect the degradation process. Other physicochemical and shape memory properties were also discussed. The achieved results are presented in a scientific way. In addition, it suited well under the scope of evaluation of biomaterials for tissue engineering. Based on excellent scientific outcomes from the present study, the paper can be accepted for publication after addressing the following points.
1. The comparison of mass loss under tensile and compressive stress predicted by empirical equation and experimental datasets should be discussed to highlight the superiority of this work.

Response：

Thanks for the comments. Empirical equations are compared with the tensile and compressive experimental datasets respectively, as shown in Fig. 5b and d. The predicted mass loss fit well with the experimental results from 0.3 MPa tensile load group and 0.6 MPa compressive group, and the determination coefficient R^2^ are 0.955 and 0.937. It shows that the empirical equation can well predict the PGD degradation under tensile or compressive stress. The above mentioned is added in the discussion at page 19 line 21.

2. Conclusion paragraph: It is a simple repetition of the results ... it adds nothing to what has already been said.
Response：

Thanks for the comment. The modified conclusion is listed in the revised manuscript at page 20 line 18 and showed as following: “The loading devices were made to explore PGD degradation under tensile and compressive load, confirming the accelerated degradation of PGD under tensile load and the suppressive degradation of PGD under compressive load. Empirical equations were obtained to describe mass loss of PGD under mechanical load, and the calculated PGD degradation results under tensile or compressive load showed a high correlation with the experimental data. A continuum damage model was designed to simulate surface erosion degradation of PGD under mechanical load, which gave a protocol for PGD implants with different geometric structures at varied mechanical conditions. There were still some limitations in this study that need to fulfill in further studies, such as an improved continuum damage simulation that concerning exposed surface area of each element, long term degradation data under mechanical load in vitro or in vivo, the polymer constitutive model in the simulation presenting shape memory properties of PGD implants between body and room temperature etc.”

Reviewer: 2
Comments to the Author
The authors investigated the effects of mechanical load on the degradation of poly(glycerol-dodecanoate) (PGD), a shape memory elastomer with a potential for use in biomedical engineering. The study aims to shed light on the relationship between mechanical load and PGD degradation, which is interesting. However, the work must be significantly improved before it can be reconsidered for publication. The detailed revision suggestions are listed as follows.
1- The study's use of an inaccurate simulation fails to represent the real physiological conditions of the body, which must be modified to support the claimed scientific merit.

Response：

Thanks for the comment. It is vital to create an accurate simulation to represent the degradation of implants in real physiological condition of the body, which is one of the key scientific question researchers working on [1] . As far as we know, there are still no studies on the PGD degradation under mechanical load, let alone simulating the surface erosion of the implants made by PGD. Lacking of relevant researches will hinder development of PGD implants in the biomedical engineering. Herein, we developed the loading devices to explore PGD degradation under tensile and compressive load, confirming the accelerated degradation of PGD under tensile load and the suppressive degradation of PGD under compressive load. Empirical equations are obtained to describe mass loss of PGD under mechanical load, and a continuum damage model is designed to simulate surface erosion degradation of PGD under mechanical load, which gave a protocol for PGD implants with different geometric structures at varied mechanical conditions. Rome was not built in a day. There are some limitations in this study that need to fulfill in further studies, such as an improved continuum damage simulation that concerning exposed surface area of each element (response for comment 7 for detail), long term degradation data under mechanical load *in vitro* or *in vivo*, the polymer constitutive model in the simulation presenting shape memory properties of PGD between body and room temperature etc. The above-mentioned content has been added to the manuscript at page 21 line 4.

2- The authors are encouraged to provide a more detailed synthesis and sample fabrication method to ensure the reproducibility of their work. Precursors and materials utilized should be clearly stated.

Response：

Thanks for the comment. As shown in Figure R2-1, detailed synthesis and sample fabrication method are added in the method at page 6 line 12. “Glycerin and dodecanedioic acid with equal molar ratios were mixed at 120 ℃ under nitrogen flow for 24 h. The product further reacted under a -0.08 MPa vacuum environment at 120 ℃ for another 24 h to obtain the PGD prepolymer. PGD sheets with 1mm or 3mm thickness were synthesized by reacting its prepolymer under vacuum condition at 120°C for 120 hours. PGD specimens were laser cut from the polymer sheets.”


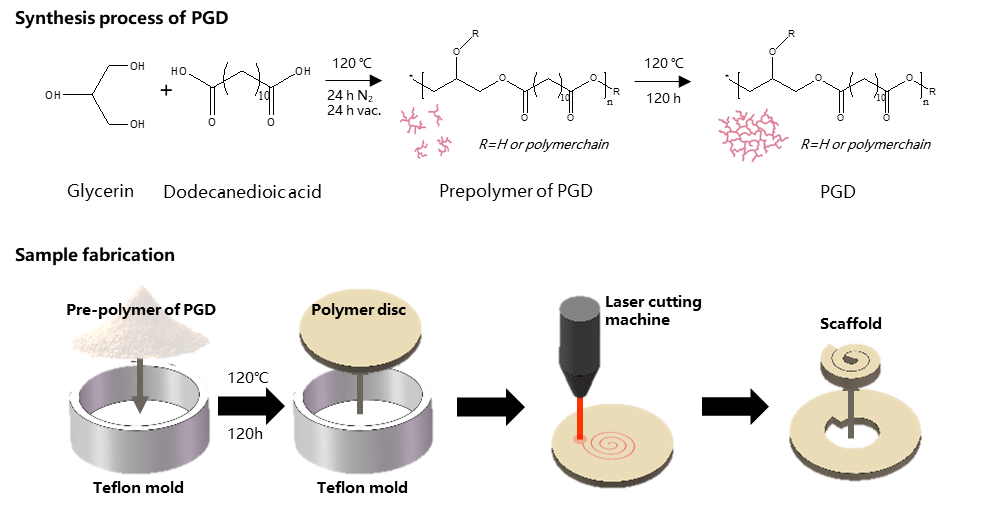


**Figure R2-1** Synthesis process and sample fabrication of PGD

3- It is imperative to consider that different tissues and organs in the body have varying mean values of the tensile/compressive stresses. The authors should clarify why the mechanical tensile/compressive loads are chosen for the degradation testing.

Response：

Thanks for the suggestion, it is helpful for us to improve the discussion in this study. The tensile/compressive stress loading ranges are determined based on stress limitation of PGD sample during 10 weeks degradation, loading conditions, and soft tissue application. In the pre-experiments, we set up several tensile load groups from 0.1 MPa to 1.0 MPa, in which the 1.0 MPa group fractured within 1 week after degradation, while most of the 0.5 MPa group samples fractured at 8 weeks after degradation. PGD groups under stress load lower than 0.4 MPa remained intact dimensions to measure material properties after 10 weeks degradation. For the load ranges during compressive degradation, 0.2 MPa-1.0 MPa was selected to match Young’s modulus of soft tissue in body, such as tendon, skin, intervertebral disc, and cartilage (Figure R2-2), considering the possible application that PGD used in the future [2, 3]. The above-mentioned content is added to the discussion at page 17 line 6 and page 18 line 2.


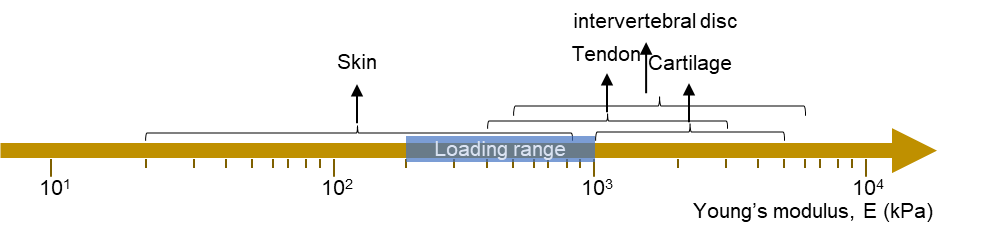


**Figure R2-2** Young’s modulus of soft tissue

4- In the section "Physicochemical characteristics of PGD polymer", the authors should review previous studies and compare the information obtained with the current study, as most of the information presented has been previously reported. For instance, please study the paper: Acta biomaterialia 122, 101-110 (2021).

Response:

Thanks for the comment. We have modified the text in the section “Physicochemical characteristics of PGD polymer” and compared the information obtained with the current studies, as following: “Thermal, shape memory, and mechanical properties of PGD polymer were investigated before degradation. Heat flow-temperature curves of PGD were measured by DSC, as shown in Fig. 3a. A typical semi-crystalline polymer was detected via an exothermic peak during cooling and an endothermic peak during heating, which was similar with previous reported PGD characteristic [4-6]. The exothermic and endothermic peak temperatures of PGD polymer in our study were 20.56±0.79℃ and 34.67±0.75℃. The crystals in the polymer chains were formed or melted when the temperature of PGD was lower or higher than the exothermic or endothermic peak temperatures respectively [6, 7]. This phenomenon gave PGD amorphous state with rubber-like property at 37℃ and glassy state with plasticity at 20℃ [8]. The phase change property between 37℃ and 20℃ could be used to program the shape of PGD polymer. As shown in Fig. 3b, the shape of PGD polymer was programed by a U-shaped mold at body temperature, and kept the U shape when the polymer temperature cooled to room temperature. The U-shaped PGD was return to its original shape when the polymer temperature back to body temperature again. Shape memory ability of PGD was able to make body temperature-triggered minimal invasive implants [9]. Mechanical properties of PGD samples at body temperature were measured via tensile and compressive conditions. Young's modulus for the tension experiment was 2.3 MPa, while Young's modulus for the compression experiment was 0.85 MPa. The Poisson's ratio of PGD was 0.47, and the material density was 1.31 g/cm^3^.”

5- The number of samples used for tensile and compressive tests needs to be specified, and the average value with standard deviation of the Young's modulus should be reported.

Response:

Thanks for the comment. Three degraded samples per group are used to measure the mechanical properties at each time point, which is labeled in the method at page 7 line 13. The average value with standard deviation of the young’s modulus is listed in the results at page 14 line 3, as shown in Table 2 and Table 3.

| Groups | Time |  |  |  |  |
| --- | --- | --- | --- | --- | --- |
|  | 2 W | 4 W | 6 W | 8 W | 10 W |
| Tens. 0.0 | 1.36±0.15 MPa | 1.07±0.09 MPa | 0.55±0.08 MPa | 0.64±0.19 MPa | 0.49±0.08 MPa |
| Tens. 0.1 | 1.54±0.09 MPa | 1.73±0.02 MPa | 0.53±0.08 MPa | 0.59±0.21 MPa | 0.69±0.05 MPa |
| Tens. 0.2 | 1.55±0.20 MPa | 1.51±0.28 MPa | 1.12±0.18 MPa | 0.56±0.05 MPa | 0.55±0.09 MPa |
| Tens. 0.3 | 1.39±0.12 MPa | 1.09±0.17 MPa | 0.59±0.05 MPa | 0.73±0.24 MPa | 0.39±0.16 MPa |
| Tens. 0.4 | 1.56±0.05 MPa | 0.86±0.19 MPa | 0.77±0.14 MPa | 0.66±0.17 MPa | 0.62±0.11 MPa |

**Table 2** Young's modulus of tensile samples after degradation

| Groups | Time |  |  |  |  |
| --- | --- | --- | --- | --- | --- |
|  | 2 W | 4 W | 6 W | 8 W | 10 W |
| Comp. 0.0 | 0.40±0.11 MPa | 0.46±0.03 MPa | 0.50±0.11 MPa | 0.35±0.04 MPa | 0.44±0.01 MPa |
| Comp. 0.2 | 0.48±0.08 MPa | 0.58±0.08 MPa | 0.59±0.12 MPa | 0.51±0.11 MPa | 0.63±0.08 MPa |
| Comp. 0.4 | 0.63±0.11 MPa | 0.44±0.05 MPa | 0.53±0.13 MPa | 0.53±0.02 MPa | 0.51±0.08 MPa |
| Comp. 0.6 | 0.67±0.10 MPa | 0.40±0.05 MPa | 0.54±0.03 MPa | 0.55±0.11 MPa | 0.42±0.11 MPa |
| Comp. 0.8 | 0.40±0.05 MPa | 0.44±0.10 MPa | 0.50±0.12 MPa | 0.49±0.06 MPa | 0.48±0.06 MPa |
| Comp. 1.0 | 0.45±0.06 MPa | 0.61±0.14 MPa | 0.52±0.16 MPa | 0.47±0.12 MPa | 0.44±0.04 MPa |

**Table 3** Young's modulus of compressive samples after degradation

6- The authors should provide an explanation for the phenomenon of oriented pores formation through degradation, which is observed in the SEM of tensile samples but not in the compression samples. Additionally, the difference in the pore morphology of the control groups without load needs to be addressed.

Response:

Thanks for the comment. Oriented pores formed in tensile samples are related to tensile stress loaded on the erosion pores of degraded samples, which are always aligned along the stress direction. The similar phenomenon is also shown in other biodegradable polyester and metals, such as PLGA and Magnesium[10, 11]. For the PGD samples under compressive load, oriented pore can hardly appear on the polymer surface when the erosion pores under compressive load. The compressed erosion pores tend to maintain the circular shape and not expand into oriented pores without tensile stresses;

The difference in the pore morphology of the control groups is related to the varied sample shape and degradation conditions during tensile/compression degradation experiments. Control group of the tensile degradation are dogbone-shaped and stretched naturally in 37℃ PBS solution during degradation. Control group of the compressive degradation are cylinder shaped and covered by the plate from top and bottom in 37℃ PBS solution during degradation. The contact surface with PBS solution is different between the two control groups, which may lead to the varied surface morphology. Besides, convection in 37℃ PBS solution may also affect micro-morphology of the polymer sample during experiments (Figure R2-3) [12]. The differences are led to varied morphology between the two control groups for the varied sample shape and degradation conditions, which do not affect the degradation results of the tensile or compressive degradation. The above mentioned is added in the discussion of the manuscript at page 19 line 1.


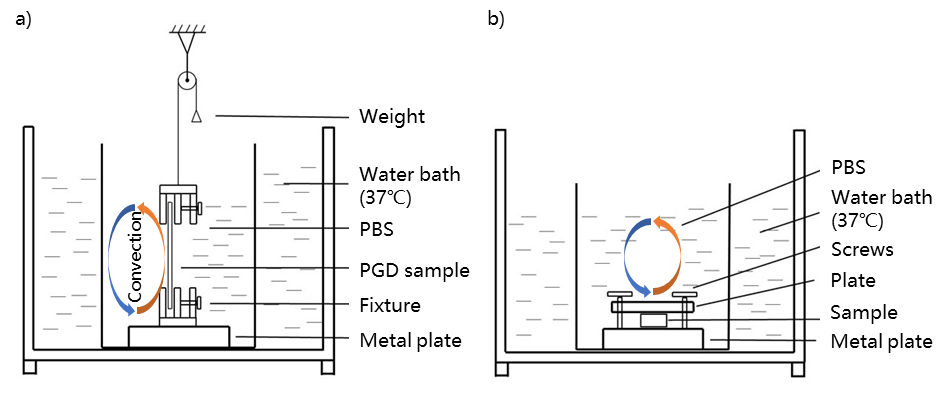


**Figure R2-3** Convection in PBS solution during tensile (a) and compressive (b) degradation

7- The inconsistency in the mass loss percentage between the control groups suggests that the shape difference of materials significantly affects the degradation rate. This finding can be utilized as a factor in the simulation to enhance its accuracy. Authors must address this issue seriously. Otherwise, the whole conclusion would be shaken. 77

Response:

Thanks for the comment. The shape difference between the control groups affects the PGD degradation rate as you mentioned, which is correlated with the surface erosion characteristics of PGD during degradation. This phenomenon is confirmed by our results as shown in Figure 4 and previous reports [13-15]. Mass loss of the PGD sample mostly occurs on its outer surface. Thus, degradation rate of PGD sample is influenced by the surface area that contact with the PBS solution. A higher contact area leads to a faster mass loss for the control groups of PGD. To evaluating the degradation between the control groups of tensile and compressive experiment, it is necessary to eliminate surface area factor among them. Herein, mass loss per unit area is employed to eliminate surface area factor between the control groups, the equation is listed as following:

Mass loss per unit area (mg/mm^2^) = (m_0_-m)/S

Where m_0_ (mg) represents the mass of PGD sample before degradation; m represents the mass of PGD sample after degradation; S (mm^2^) represents the surface area of PGD samples that contact with PBS solution. The results are shown in Figure R2-4, indicating a similar degradation of the control groups without stress load during the 10 weeks experiment. Therefore, we believe that mass loss results of tensile experiments and compressive experiments (figure 4 a&b) are comparable, and reasonable to adopt in the FEA simulation.

For the comments that concerning the influence of the shape factor in simulation, it is a very good idea. In fact, the FEA program used now can also simulate PGD implants with different shape by the continuum damage model mimicking surface erosion properties of PGD. A program concerning contact area of each element in the simulation will further improve the accuracy, and our previous works have created similar FEA program to simulate degradation of magnesium *in vivo* [16]. It still needs more degradation data to fulfill the opportunity in the future work.


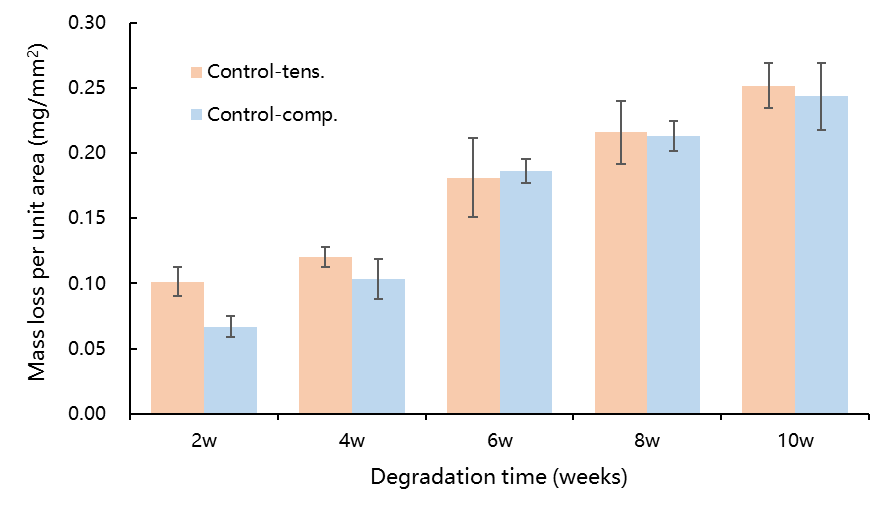


**Figure R2-4** Mass loss per unit area between control group of tensile degradation and compressive degradation.

8- The authors should provide a more comprehensive discussion and statistical analysis of the significance of the differences between mass loss percentages shown in Figures 4a and 4b.

Response:

Thanks for the comment. The promotive/inhibitory effect shown in Figure 4a and 4b on the degradation of PGD samples under tensile/compressive stress load was correlated with the degradation mechanism of the polymer. Typical surface erosion degradation is confirmed by the surface/cross-section SEM images and the linear mass loss of PGD, as shown in Figure 4. The degradation of PGD samples occurs from the outer surface where in contact with the PBS solution, thus the larger surface area of the sample the faster degradation rate it owns. The erosion pores on the outer surface of PGD sample tend to distribute along the tensile stress direction, which facilitates to expansion of the erosion pores and increase the surface area in contact with the PBS solution. This phenomenon is confirmed by the results shown in Figure 4a where the mass loss of 0.3-0.4 MPa group is significantly higher than that of control group or experimental groups with lower stress at 6 weeks after degradation, while that of 0.25 MPa group experienced the faster degradation rate than control group at 10 weeks. The erosion pores are difficult to expand under compressive stress load, instead they tend to be rounded during degradation, which leads to a reduction in the surface area of PGD sample and inhibitory degradation rate. This phenomenon is confirmed by the results shown in figure 4b where the higher compressive stress loads the slower degradation rate the PGD samples have. The above-mentioned content is added to the discussion of manuscript at page 18 line 13.

9- The authors should explain why some samples showed an increase in the cross-link density over time as shown in Figure 4f. Because the cross-linking density typically decreases with a significant mass loss as observed in most samples.

Response:

Thanks for the comment. Crosslink density of the groups in Figure 4f shows fluctuant during the 10 weeks degradation as you mentioned. This phenomenon is related to the surface erosion of PGD polymer and low degradation rate of the samples loaded with compressive stress. Linearly fitting the crosslink density - degradation time of each group is found that their crosslink densities do not change much with time, i.e., compression modulus of PGD samples do not change during 10 weeks according to Eq. 3. Sample erosion during degradation occurs on the surface of the cylinder sample in contact with the PBS solution; and it does not penetrate to the inner resulting in porous sample like bulk erosion polymer [13, 17]; the surface erosion degradation of PGD has little effect on the compression modulus of the samples. Besides, compressive stress loaded on the samples slows down the degradation, and over 90% of samples’ initial mass is remained after 10 weeks degradation. Thus, the compression modulus/crosslink density of samples does not change much. The above mentioned is added to the discussion of manuscript at page 18 line 9.

Review Editor
Comments to the Author:
Please revise the graphic abstract.
Please notice that the GA should be prepared and displayed at the actual size of 1.4 inches (height) × 3.3 inches (width) or 3.6 cm × 8.4 cm following the Abstract in the submitted manuscript file.
Minimum resolutions are 300 d.p.i. for colour or tone images, and 600 d.p.i. for line drawings. Preferred font type of the texts in the GA is Arial 8-16 points (smaller will not be legible). Try not to use the distracting and cluttering elements as much as possible. Please do not enlarge the GA, because the image will eventually appear on the first page of the published PDF file in the specified size above. For examples of graphical abstracts, please refer to the published papers since 2022.

Response:

Thanks for the comment. The graphic abstract has been modified following the rules above listed. Font size in the figure is enlarged to make them more visible.

**References**

1. Wang L, Ding X, Feng W, Gao Y, Zhao S, Fan Y. Biomechanical study on implantable and interventional medical devices. *Acta Mechanica Sinica* 2021;37:875-894.

2. Sachot N, Engel E, Castaño O. Hybrid organic-inorganic scaffolding biomaterials for regenerative therapies. *Current Organic Chemistry* 2014;18:2299-2314.

3. Akman R, Ramaraju H, Verga A, Hollister SJ. Multimodal 3D printing of biodegradable shape memory elastomer resins for patient specific soft tissue repair. *Applied Materials Today* 2022;29:101666.

4. Migneco F, Huang YC, Birla RK, Hollister SJ. Poly(glycerol-dodecanoate), a biodegradable polyester for medical devices and tissue engineering scaffolds. *Biomaterials* 2009;30:6479-84.

5. Solorio LD, Bocks ML, Hollister SJ. Tailoring the physicochemical and shape memory properties of the biodegradable polymer poly(glycerol dodecanoate) via curing conditions. *Journal of Biomedical Materials Research Part A* 2017;105:1618-1623.

6. Zhang C, Deng H, Kenderes SM, Su JW, Whittington AG, Lin J. Chemically Interconnected Thermotropic Polymers for Transparency-Tunable and Impact-Resistant Windows. *ACS Appl Mater Interfaces* 2019;11:5393-5400.

7. Zhang C, Cai D, Liao P, Su J-W, Deng H, Vardhanabhuti B, Ulery BD, Chen S-Y, Lin J. 4D Printing of shape-memory polymeric scaffolds for adaptive biomedical implantation. *Acta biomaterialia* 2021;122:101-110.

8. Ramaraju H, Massarella D, Wong C, Verga AS, Kish EC, Bocks ML, Hollister SJ. Percutaneous delivery and degradation of a shape memory elastomer poly (glycerol dodecanedioate) in porcine pulmonary arteries. *Biomaterials* 2023;293:121950.

9. Ramaraju H, Akman RE, Safranski DL, Hollister SJ. Designing biodegradable shape memory polymers for tissue repair. *Advanced Functional Materials* 2020;30:2002014.

10. Gao Y, Wang L, Li L, Gu X, Zhang K, Xia J, Fan Y. Effect of stress on corrosion of high-purity magnesium in vitro and in vivo. *Acta Biomaterialia* 2019;83:477-486.

11. Guo M, Chu ZW, Yao J, Feng WT, Wang YX, Wang LZ, Fan YB. The effects of tensile stress on degradation of biodegradable PLGA membranes: A quantitative study. *Polymer Degradation and Stability* 2016;124:95-100.

12. Fang Q, Ye F, Yang X. Hierarchical Morphology of Polymer Blend Films Induced by Convection-Driven Solvent Evaporation. *Langmuir* 2018;34:5551-5557.

13. Wan L, Lu L, Zhu T, Liu Z, Du R, Luo Q, Xu Q, Zhang Q, Jia X. Bulk Erosion Degradation Mechanism for Poly (1, 8-octanediol-co-citrate) Elastomer: An In Vivo and In Vitro Investigation. *Biomacromolecules* 2022;23:4268-4281.

14. Gopferich A, Tessmar J. Polyanhydride degradation and erosion. *Adv Drug Deliv Rev* 2002;54:911-31.

15. Ramaraju H, Solorio LD, Bocks ML, Hollister SJ. Degradation properties of a biodegradable shape memory elastomer, poly(glycerol dodecanoate), for soft tissue repair. *PLoS One* 2020;15:e0229112.

16. Gao Y, Wang L, Gu X, Chu Z, Guo M, Fan Y. A quantitative study on magnesium alloy stent biodegradation. *J Biomech* 2018;74:98-105.

17. Yang XD, Zhang WR, Yao J, Sun AQ, Gao YM, Guo M, Fan YB. The differences between surface degradation and bulk degradation of FEM on the prediction of the degradation time for poly (lactic-co-glycolic acid) stent. *Computer Methods in Biomechanics and Biomedical Engineering* 2022;25:65-72.
